# Stabilization of Poly (β-Amino Ester) Nanoparticles for the Efficient Intracellular Delivery of PiggyBac Transposon

**DOI:** 10.3390/bioengineering8020016

**Published:** 2021-01-20

**Authors:** Tina Rodgers, Nicolas Muzzio, Caleb Watson, Gabriela Romero

**Affiliations:** Department of Biomedical Engineering and Chemical Engineering, University of Texas at San Antonio, One UTSA Circle, San Antonio, TX 78249, USA; tina.rodgers@utsa.edu (T.R.); nicolas.muzzio@utsa.edu (N.M.); caleb.watson378@gmail.com (C.W.)

**Keywords:** nanoparticles, plasmid delivery, gene therapies, non-viral vectors, crosslinking, layer-by-layer

## Abstract

The administration of gene-editing tools has been proposed as a promising therapeutic approach for correcting mutations that cause diseases. Gene-editing tools, composed of relatively large plasmid DNA constructs that often need to be co-delivered with a guiding protein, are unable to spontaneously penetrate mammalian cells. Although viral vectors facilitate DNA delivery, they are restricted by the size of the plasmid to carry. In this work, we describe a strategy for the stable encapsulation of the gene-editing tool piggyBac transposon into Poly (β-amino ester) nanoparticles (NPs). We propose a non-covalent and a covalent strategy for stabilization of the nanoformulation to slow down release kinetics and enhance intracellular delivery. We found that the formulation prepared by covalently crosslinking Poly (β-amino ester) NPs are capable to translocate into the cytoplasm and nuclei of human glioblastoma (U87MG) cells within 1 h of co-culturing, without the need of a targeting moiety. Once internalized, the nanoformulation dissociates, delivering the plasmid presumably as a response to the intracellular acidic pH. Transfection efficiency is confirmed by green fluorescence protein (GFP) expression in U87MG cells. Covalently stabilized Poly (β-amino ester) NPs are able to transfect ~55% of cells causing non-cytotoxic effects. The strategy described in this work may serve for the efficient non-viral delivery of other gene-editing tools.

## 1. Introduction

In recent years gene-editing therapies have emerged as a promising avenue for the treatment of various diseases ranging from genetic disorders [1] and infections [2,3,4] to cancer [5,6,7]. Particularly for cancer treatment, gene-editing therapy works by specifically inhibiting or editing the expression of the gene of interest. Small interfering RNA (siRNA) technologies are based on the silencing of selected genes [8], while new gene-editing tools such as meganucleases, zinc finger nucleases (ZFN) [9,10], transcription activator-like effector nucleases (TALEN) [11,12], clustered regularly interspaced short palindromic repeats (CRISPR/Cas9) [13] or the piggyBac transposon/transposase [14] apply a “cut-and-paste” approach to replace specific DNA sequences. Despite the breakthrough therapeutic possibilities, a major challenge for clinical translation of gene-editing therapies is to find a suitable and safe vehicle that ensures the intracellular delivery of nucleic acids as free exogenous plasmids are not able to spontaneously penetrate into mammalian cells [15,16,17,18].

Currently, the most common delivery systems utilized in gene therapy are viral vectors, which use the virus capsid as a means to introduce genetic materials. Viral vectors are very effective in gene delivery as they are built upon the natural ability of viruses to infect cells while being modified to be used in therapy [19,20,21]. However, viral vectors pose a number of disadvantages: (i) viruses are limited to a cargo size of ~4.5–5 kb, making them unsuitable for gene-editing therapies, where plasmid vectors are larger than 6 kb and often need to be co-delivered with a guiding protein [21,22]; (ii) virus vectors could potentially induce immunogenic and mutagenic responses [21]; and (iii) virus vectors are difficult to produce on a large scale [23]. To overcome these clinical challenges, non-viral approaches using lipids [24,25], polymers [26,27,28,29], or inorganic nanomaterials [30,31] have been proposed as alternative vectors. Although none of these alternative vectors have been able to match the competence of a virus due to low cell-type specificity and failure to escape from the endosome, synthetic carriers offer the possibility for chemical and macromolecular design to improve their capacity of incorporation of nucleic acids and to gain control over intracellular trafficking [32].

The most efficient non-viral vectors for gene therapy are based on cationic polyelectrolytes and lipids [33,34,35]. Positive charges through the protonation or alkylation of the amine groups of both, polyelectrolytes and lipids, are essential for complexing negatively-charged nucleic acids and delivering them into the cell. Positive charges are also necessary for the translocation of the nucleic acid from the endosome to the cytoplasm as they can disrupt lipid membranes by interacting with phospholipids, which are negatively-charged. Osmotic endosomal swelling can also be induced through protonation of amine groups [36]. Although positive charges are very important for the encapsulation and efficient delivery of nucleic acids, they are often responsible for the cytotoxicity of non-viral vectors.

Cationic polymers synthesized from Poly(β-amino esters) (PBAEs) have attracted enormous attention for their application in controllable and programmable gene delivery because they are biocompatible [37] and biodegradable (via hydrolysis of ester groups) [38]. The tertiary amine groups present in PBAEs can electrostatically interact with negatively charged nucleic acids to form nanoparticles. Moreover, PBAEs are pH-sensitive as the amino groups in the polymer chain undergo phase transition upon the change of surrounding pH [39], facilitating the intracellular delivery of genes. A wide range of polymers and co-polymers composed of PBAEs have been reported demonstrating their versatility in engineering their physical, chemical, and mechanical properties [40]. However, very few studies have been done using co-polymers of PBAEs for gene-editing tool delivery.

**Figure 1:**
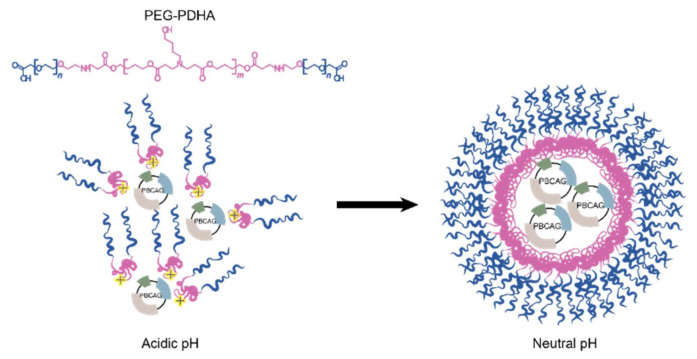
Schematic representation of the gene-editing tool piggyBac transposon (PBCAG) plasmid encapsulation through complexation with amine groups in the poly (1,4-butanediol)–diacrylate-β–hydroxyamylamine) (PDHA) polymer block.

In this work, we study the fabrication of nanoparticles (NPs) composed of the PBAE co-polymer poly (ethylene glycol)–block–poly(1,4-butanediol)–diacrylate-β,–hydroxyamylamine–block–poly(ethylene glycol) (PEG-PDHA) for the stable encapsulation and intracellular delivery of the gene-editing tool piggyBac transposon (PBCAG). PEG was conjugated to PDHA to improve systemic circulation time and to decrease PDHA charge-associated cytotoxicity. Although PEG-PDHA NPs display a relatively small and narrow size distribution, and the PEG chains are at high density forming a thick shell layer on the NPs surface (Figure 1), we found that PBCAG releases very fast from these NPs, hindering gene delivery. We studied the stabilization of PEG-PDHA NPs through a non-covalent and a covalent route. Non-covalent stabilization was done by creating a coating on the PEG-PDHA NPs surface through the layer-by-layer assembly of biopolyelectrolytes. Covalent stabilization was carried out through chemical crosslinking between polymer chains. We found that covalent crosslinking provides the most efficient option for PEG-PDHA NPs stabilization, resulting in the high-efficiency intracellular delivery of PBCAG.

## 2. Materials and Methods

### 2.1. Materials

1,4-butanediol diacrylate, 4-amino-1-butanol, dimethyl sulfoxide (DMSO), triethylamine (TEA), pyridine (Py), dialysis bag 10 kDa, sodium chloride (NaCl), poly-L-lysine hydrobromide (PLL), poly-L-γ-glutamate sodium salt (PGA), 3-(4,5-dimethylthiazol-2-yl)-2,5-diphenyltetrazolium bromide (MTT assay), DAPI, phosphate buffer Saline (PBS), Eagle’s minimum essential medium (EMEM), fetal bovine serum (FBS), ampicillin sodium salt and opti-MEM 1 reducing serum media were purchased from Sigma Aldrich. NH_2_-PEG-COOH was purchased from Laysan Bio. The human glioblastoma (U87MG) cell line was purchased from ATCC. Rhodamine B Isothiocyanate was purchased from CHEM-IMPEX International Inc. Eosin-5-Isothiocyanate was obtained from Chemodex. Ultrapure Agarose, Lipofectamine 3000, lysogeny broth (LB), and SYBR Safe DNA gel stain were acquired from Thermo Fisher Scientific. Tris/Borate/EDTA (TBE) Buffer was obtained from Alfa Aesar. Plasmid PiggyBac transposon (PBCAG-eGFP, 6.34 kb) was purchased from Addgene as bacterial agar stab. Bacterial amplification was performed using a sterile LB solution which contained ampicillin and was purified utilizing an endotoxin-free DNA purification kit from QIAGEN.

### 2.2. Synthesis of PEG-PDHA

PEG-PDHA was synthesized utilizing the Michael addition polymerization technique [37]. For the first step, poly(1,4-butanediol)-diacrylate-β 5-hydroxyamylamine (PDHA) was synthesized by combining 1,4-butanediol diacrylate (8.23 mmol) with 4-amino-1-butanol (7.48 mmol) in a 1.1:1 (diacrylate:amine) molar ratio. The reaction was heated to 90 °C for 45.5 h under magnetic stirring and N_2_ gas flow. After 45.5 h, the reaction product was dissolved in DMSO and sonicated with heat. The mixture was then centrifuged, and the dissolved portion was decanted and dried in a rotary evaporator. To verify the purity of the PDHA, an aliquot of the product was removed and characterized via proton nuclear magnetic resonance (^1^H-NMR). PDHA was dissolved again in DMSO and stored at -20 °C until further needed. For the second step, NH_2_-PEG-COOH (0.05 mmol) was dissolved in DMSO. This solution was sonicated to make sure all NH_2_-PEG-COOH was dissolved completely. The solution was placed under magnetic stirring and pyridine (6.18 mmol) and TEA (3.59 mmol) were added. The PDHA previously synthesized was added dropwise to the solution every twelve minutes. The molar ratio of the PDHA to NH_2_-PEG-COOH used was 1:2. The reaction was carried out for 24 h to produce the COOH terminated PEG-PDHA. PEG-PDHA was purified through dialysis against millipore water for 72 h. After purification, PEG-PDHA was lyophilized and stored at −20 °C.

### 2.3. PEG-PDHA Characterization

A ^1^H-NMR was performed to verify the purity of the PEG-PDHA polymer. PEG-PDHA nanoparticles were formed by resuspending the polymer in PBS. The ζ-potential and hydrodynamic diameter were measured utilizing dynamic light scattering (Malvern Nano Zetasizer). Every ζ-potential measurement was performed using Millipore water at 25 °C and a cell drive voltage of 30 V, utilizing the monomodal analysis model. Transmission electron microscopy was performed to further characterize the particles.

### 2.4. Plasmid Encapsulation with PEG-PDHA

PEG-PDHA was dissolved in 0.15 M NaCl and PBCAG-eGFP was added carefully to the PEG-PDHA solution at different polymer/plasmid molar ratios of: 18, 9, 3.6, 1.8, 0.9, 0.36 and 0.18. This solution was placed on an orbital shaker for 45 min at 4 °C allowing for plasmid encapsulation. After the encapsulation period, particles were centrifuged at 18,000 rpm for 5 min. Supernatant was removed and the pellet was resuspended in PBS at pH 4 and 7. The sample was sonicated for complete resuspension and placed back on the orbital shaker.

### 2.5. Plasmid Encapsulation with Layer-by-Layer

PEG-PDHA was dissolved in 0.15 M NaCl and PBCAG-eGFP was carefully added to the PEG-PDHA solution in the same fashion as described above. Then, the surface of the PEG-PDHA NPs was coated with layers of the biopolyelectrolytes PGA and PLL assembled through the layer-by-layer technique. Briefly, after the first centrifugation, the supernatant was removed, and the NPs pellet was resuspended in a solution composed of 0.5 mg PGA in 0.15 M NaCl at pH 4. The NPs were placed back on the orbital shaker for 30 min to allow for the electrostatic interaction between the PEG-PDHA and the PGA. NPs were again centrifuged at 18,000 rpm for 5 min at 4 °C. The supernatant was decanted, and the pellet was resuspended in a solution composed of 0.5 mg PLL in 0.15 M NaCl pH 4. NPs were placed back on the orbital shaker for 30 min to allow for the electrostatic interaction between PGA and the PLL. The process was repeated to assemble two bilayers of PGA/PLL. Finally, the NPs were centrifuged at 18,000 rpm for 5 min at 4 °C. The supernatant was decanted, and the pellet was resuspended with PBS at pH 4 and 7.

### 2.6. Plasmid Encapsulation and Crosslinking

PEG-PDHA was dissolved in 0.15 M NaCl and PBCAG-eGFP was added carefully to the PEG-PDHA solution in the same fashion as described above. After centrifugation, the PEG-PDHA polymer was crosslinked by incubating the NPs in a solution containing 10 mM N-hydroxysuccinimide (NHS) and 10 mM 1-ethyl-3-(3-dimethylaminopropyl) carbodiimide hydrochloride (EDC) at pH 5.6 for 1 h. Then, the pH was adjusted to approximately 8.6 and left overnight for conjugation at 4 °C under an orbital shaker. After 24 h, the sample was centrifuged at 18,000 rpm for 5 min at 4 °C. The supernatant was again discarded, PBS was added to the pellet and sonicated until the pellet was dissolved. The sample was again placed on an orbital shaker at 4 °C until needed.

Characterization was performed on the NPs at each step of functionalization. Size and surface charge were characterized by dynamic light scattering. Gel electrophoresis was performed to verify plasmid encapsulation for all plasmid encapsulation samples, with a 1.0% agarose gel using TBE running buffer. Gel imaging was done using a Lonza FlashGel Imaging unit and using SYBR Safe for plasmid staining. UV-spectroscopy was performed to quantify plasmid encapsulation.

### 2.7. Plasmid Release Kinetics

The different PEG-PDHA NPs encapsulating PBCAG-eGFP were placed in an incu-shaker at 37 °C and 150 rpm. At the time intervals of 0, 1, 3, 24, 48, and 72 h, aliquots were removed and centrifuged at 18,000 rpm for 5 min. Released plasmid was quantified by UV spectroscopy of the supernatant, while encapsulated plasmid was characterized in the NPs pellet by gel electrophoresis and dynamic light scattering.

### 2.8. PEG-PDHA NPs Cellular Uptake

For these studies, PEG-PDHA NPs were labeled with either Rhodamine B isothiocyanate or Eosin isothiocyanate by covalently binding the isothiocyanate to the carboxylic end groups of PEG [41]. The U87MG cell line was cultured in EMEM with 10% FBS and cells were incubated at 37 °C in 5% CO_2_. After cell confluency was approximately 80%, TripLE was used to detach all the cells. A 96-well plate was used for the experiment and 15,000 cells were seeded per well with 200 µL of EMEM. 24 h after incubation, U87MG cells were exposed to PEG-PDHA NPs labeled with Rhodamine B, which were added to the media at concentrations of 5 µg µL^−1^. At different time intervals, 0, 0.5, 1, 2, 5, and 24 h, the cells were cleaned twice with PBS, detached with TripLe and flow cytometry was performed on them. The fluorescence threshold was established using a control sample, which consisted of the intact U87MG cells that were not exposed to the NPs. Parameters for every measurement were set the same for all samples, and every run was set to count 10,000 events. The total percentage of cells that contain the fluorescent labeled NPs was obtained within the area that corresponds to the higher intensities above the threshold.

A Leica TCS SP8 Confocal Microscope was used to image cells co-cultured with the NPs labeled with Eosin. Cell samples were fixed with 4% paraformaldehyde in PBS and permeabilized with 0.1% Triton X-100 for 5 min. TRITC-conjugated phalloidin and 4′,6-diamidino-2-phenylindole (DAPI) were used for F-actin and nucleus staining, respectively.

### 2.9. In Vitro Transfection Efficiency

U87MG cells were planted in 96-well plates as described above. 24 h after culturing, U87MG cells were exposed to PEG-PDHA NPs in 20 µL of Opti-MEM at concentrations of 5 and 10 µg µL^−1^ of PBCAG-eGFP per well. Lipofectamine 3000 was used as the positive control to compare the transfection efficacy against the PEG-PDHA NPs. Flow cytometry was performed on the cells to quantify the GFP expression utilizing a BD Accuri C6 Plus flow cytometer. A fluorescence threshold was set utilizing U87MG intact cells that were not exposed to NPs. The parameters for measurements were set the same for all samples and each run was set to count 10,000 events. The total count of cells that expressed GFP was determined from the area corresponding to higher intensities than the threshold. Confocal microscopy was utilized to qualitatively characterize the cells expressing GFP using a Leica TCS SP8 Confocal Microscope.

### 2.10. PEG-PDHA NP Toxicity

U87MG cell viability was verified utilizing an MTT assay. Cells were seeded in 96-well plates as described above. After 24 h, Lipofectamine 3000 or PEG-PDHA NPs, both formulated with 5 µg µL^−1^ of PBCAG-eGFP in 20 µL of Opti-MEM, were added to each well. Cell viability was measured for 3 consecutive days. Each day 50 µL of MTT solution (5 mg mL^−1^ in 1X PBS) was added to every well. After 1 h, the media was carefully removed and 200 µL DMSO was added. The absorbance at 570 nm was measured utilizing a plate reader. The percentage of cell viability was calculated by normalizing the absorbance of the control (intact cells were set to 100%).

## 3. Results

### 3.1. Synthesis and Characterization of PEG-PDHA

PEG-PDHA was synthesized utilizing the previously reported Michael addition polymerization technique [37]. PEG-PDHA structure and purity were verified by ^1^H-NMR (Figure S1). It was found that the previously reported polymerization protocol by Tang et. al., is highly reproducible for the synthesis of PEG-PDHA with a conversion efficiency >80%. As reported previously and consistent with our NMR results, the conjugation reaction between PEG and PDHA does not form undesired side products, and the ~20% impurities are residual unconjugated carboxyl terminated PEG and PDHA [37,42,43].

### 3.2. Fabrication of PEG-PDHA NPs

**Figure 2:**
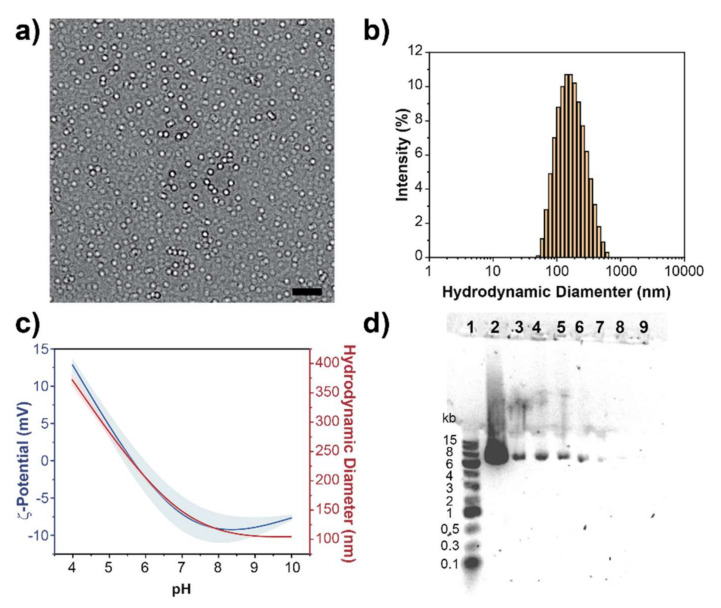
Poly (ethylene glycol)–block–poly(1,4-butanediol)–diacrylate-β,–hydroxyamylamine–block–poly(ethylene glycol) (PEG-PDHA) nanoparticles (NPs) characterization. (**a**) Transmission electron microscopy (TEM) image of PEG-PDHA NPs, scale bar = 500 nm. (**b**) Hydrodynamic size distribution of PEG-PDHA NPs in PBS. (**c**) PEG-PDHA NPs ζ-potential and hydrodynamic diameter as a function of pH. Solid lines indicate mean value and shadows indicate standard deviation. (**d**) Gel Electrophoresis PEG-PDHA NPs encapsulating PBCAG at different molar ratios. Well 1: 1 kb Plus DNA ladder, Well 2: PBCAG (6.34 kb), Well 3–9: Polymer/plasmid at molar ratios of 18, 9, 3.6, 1.8, 0.9, 0.36 and 0.18, respectively.

Fabrication of PEG-PDHA NPs was done by resuspending PEG-PDHA in PBS at concentrations above the critical micelle concentration of PEG-PDHA (CMC_PEG-PDHA_ = 0.015 mg mL^−1^) [44,45]. Size and morphology of PEG-PDHA NPs were characterized by transmission electron microscopy (TEM) and by dynamic light scattering (DLS) (Figure 2). TEM images show that the PEG-PDHA NPs have a homogenous spherical morphology with a diameter of 143.9 ± 29 nm (Figure 2a). DLS characterization exhibited that PEG-PDHA NPs display a hydrodynamic diameter of 150.1 ± 2 nm with a narrow size distribution (PDI = 0.184) (Figure 2b). Both the diameters measured by TEM and by DLS were consistent. The surface charge of PEG-PDHA NPs was characterized by ζ-Potential measurements at different pH values to confirm the sensitivity of the PDHA polymer block (Figure 2c). Consistent with previously reported studies, PDHA protonation occurs at pH < 5.6, where the polymer forms bigger and unstable aggregates with a diameter above 250 nm and positive ζ-Potential. At physiological pH, PEG-PDHA stable NPs display a slightly negative ζ-Potential of −7.86 ± 2 mV due to the carboxylic end groups of the PEG polymer block.

### 3.3. Plasmid Encapsulation into PEG-PDHA NPs

We studied the encapsulation of the PBCAG plasmid (Addgene PBCAG-eGFP) into PEG-PDHA NPs at different molar ratios to produce a formulation with the highest encapsulation efficiency. PEG-PDHA NPs were prepared by first complexing with PBCAG at low pH, when the amine groups in PDHA are most positive, to facilitate interaction between the polymer and the plasmid. Complexation was tested for the polymer/plasmid molar ratios of: 18, 9, 3.6, 1.8, 0.9, 0.36 and 0.18. Complexation was allowed for forty-five minutes under orbital shaking at 4 °C to enhance encapsulation. After, the pH of the formulation was adjusted to 7. Gel electrophoresis was employed to verify encapsulation and encapsulation efficiency (Figure 2d). DNA migration in the gel was indicative of encapsulation failure. It was found that the molar ratio of 0.36 PEG-PDHA/PBCAG was the most efficient for encapsulation. At these conditions, PEG-PDHA NPs encapsulating PBCAG display a hydrodynamic size of 236.9 ± 10 nm (PDI = 0.192) and a ζ-potential of −6.72 ± 1 mV (Figure S2). The hydrodynamic size increased ~80 nm after PBCAG encapsulation, while the surface charge did not significantly change, indicating possible PBCAG encapsulation.

### 3.4. PBCAG Release Kinetics from PEG-PDHA NPs

**Figure 3:**
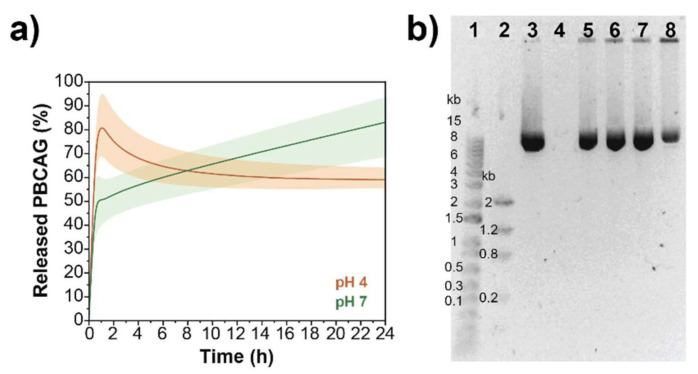
PBCAG release kinetics from PEG-PDHA NPs. (**a**) PBCAG release over a period of 24 h from PEG-PDHA NPs at 37 °C in acid and neutral environments. (**b**) Representative gel electrophoresis of PBCAG release from PEG-PDHA NPs. Well 1: 1 kb DNA ladder, Well 2: low DNA mass ladder, Well 3: PBCAG (6.34 kb), Well 4–8: PEG-PDHA NPs encapsulating PBCAG after 0, 0.5, 1, 3, and 24 h incubation at 37 °C, respectively.

A release kinetic study was performed to determine the release rate of plasmid from the polymer and the stability of the nanoformulation. PEG-PDHA nanoformulation encapsulating PBCAG at a molar ratio of 0.36 polymer/plasmid was incubated at 37 °C, at pH = 7 or 4. Aliquots of each sample were taken at different incubation times (0, 1, 3, 24, and 48 h). Aliquots were centrifuged to remove the polymer particles, and the released DNA was quantified from the supernatant by UV-spectroscopy and gel electrophoresis (Figure 3a,b and Figure S3a). Total DNA encapsulated into the NPs was quantified by UV-spectroscopy by disintegrating the nanoformulation at t = 0 with 5% SDS solution. The percentage of DNA released was calculated by referencing to the total amount of DNA encapsulated. We found that although PEG-PDHA NPs encapsulate PBCAG at very high efficiency, this nanoformulation released all DNA within the first 2 h. Using PEG-PDHA at low pH, where the complex formed with PBCAG should be stronger, it did not improve PBCAG release kinetics.

#### 3.4.1. PEG-PDHA NPs Stabilization by Layer-by-Layer Surface Engineering

**Figure 4:**
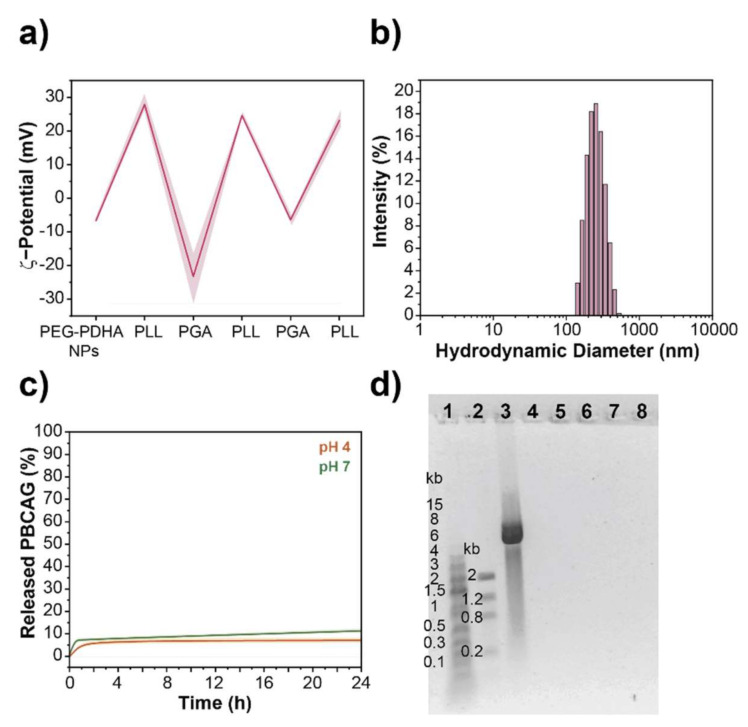
Layer-by-layer engineered PEG-PDHA nanoformulation. (**a**) The ζ-potential of PEG-PDHA NPs during layer-by-layer assembly process. (**b**) Hydrodynamic size distribution of layer-by-layer engineered PEG-PDHA nanoformulation. (**c**) Release kinetics of PBCAG from layer-by-layer engineered PEG-PDHA nanoformulation. (**d**) Representative gel electrophoresis of PBCAG release from layer-by-layer engineered PEG-PDHA nanoformulation. Well 1: 1 kb DNA ladder, Well 2: low DNA mass ladder, Well 3: PBCAG (6.34 kb), Well 4–8: PEG-PDHA NPs encapsulating PBCAG after 0, 0.5, 1, 3, and 24 h incubation at 37 °C, respectively.

To stabilize PEG-PDHA nanoformulation for PBCAG, we investigated functionalization of NPs by non-covalent and covalent routes. First, we studied the non-covalent functionalization of PEG-PDHA NPs through the self-assembly of oppositely charged biopolyelectrolytes following the layer-by-layer technique. After fabrication and PBCAG encapsulation, NPs were coated with four biopolyelectrolyte layers of poly(L-lysine) (PLL) and poly(γ-glutamate) (PGA). After each layer was assembled, NPs were washed with PBS to remove the excess polymer. ζ-potential measurements were used to confirm the assembly of each layer (Figure 4a). The ζ-potential oscillated between +28 mV and −23 mV after the assembly of PLL or PGA, respectively. After the assembly, PEG-PDHA NPs encapsulating PBCAG displayed a ζ-potential of 25.6 ± 3 mV with a hydrodynamic diameter of 252.9 ± 8 nm (PDI = 0.1) (Figure 4b). Likewise, for unmodified PEG-PDHA NPs, a release kinetics study was performed to determine the delivery rate of PBCAG from the layer-by-layer engineered nanoformulation. (Figure 4c,d and Figure S3b). We found that layer-by-layer engineered PEG-PDHA NPs do not release PBCAG within 24 h at 37 °C, neither at physiological pH nor at acidic pH.

#### 3.4.2. PEG-PDHA NPs Stabilization by Crosslinking

**Figure 5:**
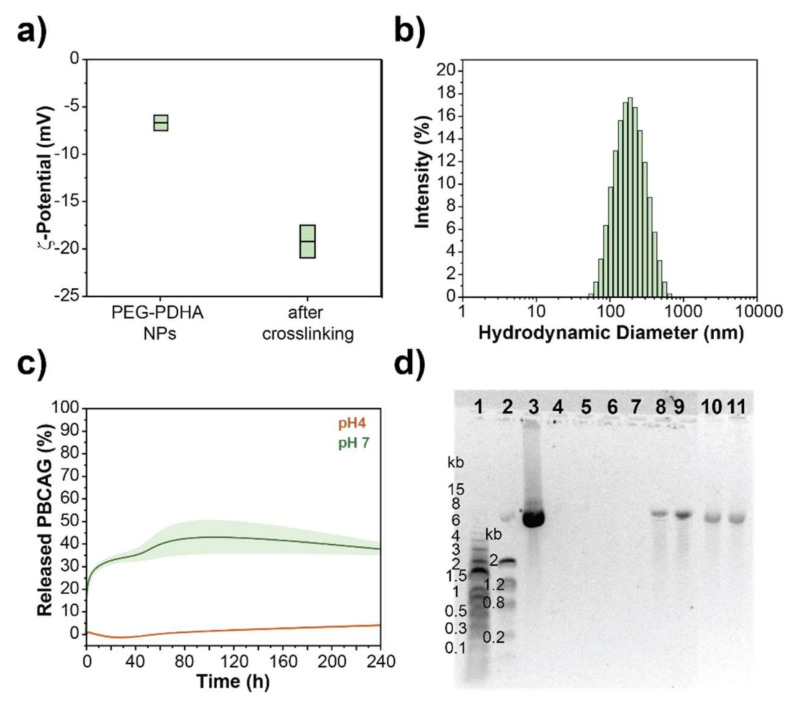
PEG-PDHA crosslinked nanoformulation. (**a**) The ζ-potential of PEG-PDHA NPs before and after crosslinking. (**b**) Hydrodynamic size distribution of PEG-PDHA crosslinked nanoformulation. (**c**) Release kinetics of PBCAG from crosslinked PEG-PDHA nanoformulation. (**d**) Representative gel electrophoresis of PBCAG release from PEG-PDHA crosslinked nanoformulation. Well 1: 1 kb DNA ladder, Well 2: low DNA mass ladder, Well 3: PBCAG, Well 4–11: PEG-PDHA NPs encapsulating PBCAG after 0, 0.5, 1, 3, 24, 48, 72 and 240 h incubation at 37 °C, respectively.

We also investigated the stabilization of PEG-PDHA nanoformulation for PBCAG through covalent crosslinking between PEG-PDHA polymer chains. After fabrication and PBCAG encapsulation, NPs were treated with 10 mM NHS/EDC solution to crosslink residual carboxylic acid end groups of PEG and the amine groups of PDHA chains within the formed complex. Crosslinking was corroborated by a decrease in the ζ-potential to −19.2 ± 2 mV (Figure 5a). Crosslinked PEG-PDHA nanoformulation displayed a hydrodynamic diameter of 177.1 ± 5 nm (PDI = 0.126) (Figure 5b). PBCAG release from crosslinked nanoformulation was studied in PBS at 37 °C. PBCAG release from crosslinked PEG-PDHA NPs in PBS was significantly slower than for unmodified PEG-PDHA nanoformulation and after 10 days only 40% of the encapsulated PBCAG was released (Figure 5c,d). At acidic pH, where the strongest complex is formed between the polymer and PBCAG, we did not detect any PBCAG release within 10 days (Figure S3c). Our results show that crosslinking of the nanoformulation can slow down the release kinetics of PBCAG in PBS.

### 3.5. PEG-PDHA NPs Cellular Uptake

**Figure 6:**
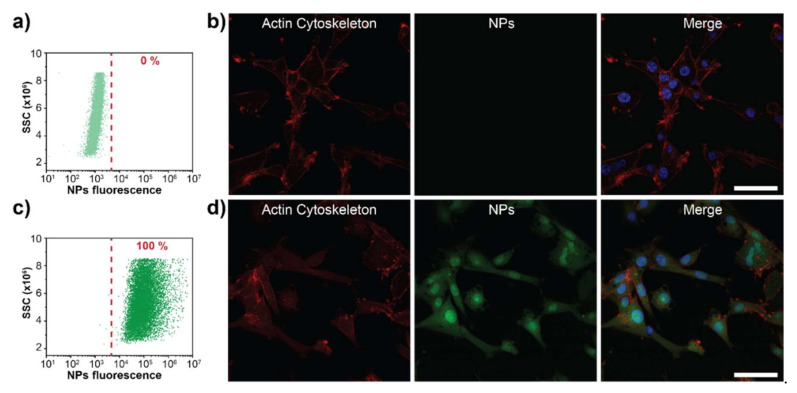
PEG-PDHA NPs uptake by U87MG cells. (**a**) Flow cytometry and (**b**) confocal images of intact U87MG cells. (**c**) Flow cytometry and (**d**) confocal images of U878MG cells co-cultured for 1 h with PEG-PDHA NPs. TRITC-conjugated phalloidin was used to stain for actin cytoskeleton (red), Eosin isothiocyanate was used to label PEG-PDHA NPs (green) and 4′,6-diamidino-2-phenylindole (DAPI) was used to stain the nuclei (blue). Scale bar = 50 μm.

Another important factor in the design of nanocarriers for gene delivery is cellular uptake rate. The nanocarrier must protect the cargo from degradation while in circulation, and it should be efficient in entering the cell and delivering the cargo intracellularly. We studied PEG-PDHA NPs cellular uptake using the human glioblastoma cell line U87MG. PEG-PDHA NPs were fluorescently labeled with Rhodamine B to study cellular uptake by flow cytometry and with Eosin Isothiocyanate to characterize cell uptake through confocal microscopy. Flow cytometry results showed that PEG-PDHA NPs are either taken up or attached to the cell membrane of U87MG cells within one hour of co-culturing (Figure 6a,c). Internalization of PEG-PDHA NPs into U87MG cells was confirmed by confocal microscopy (Figure 6b,d). The actin cytoskeleton and nuclei of cells were stained to facilitate co-localization. PEG-PDHA NPs were found to be localized in the cell cytoplasm. Interestingly, PEG-PDHA NPs were also found in the cell nucleus. Confocal images of U87MG cells incubated with PEG-PDHA NPs labeled with Rhodamine B are shown in Figure S4. No difference in cellular uptake is observed when NPs are labeled either with Eosin or Rhodamine B.

### 3.6. In Vitro Transfection Efficiency of PEG-PDHA NPs

**Figure 7:**
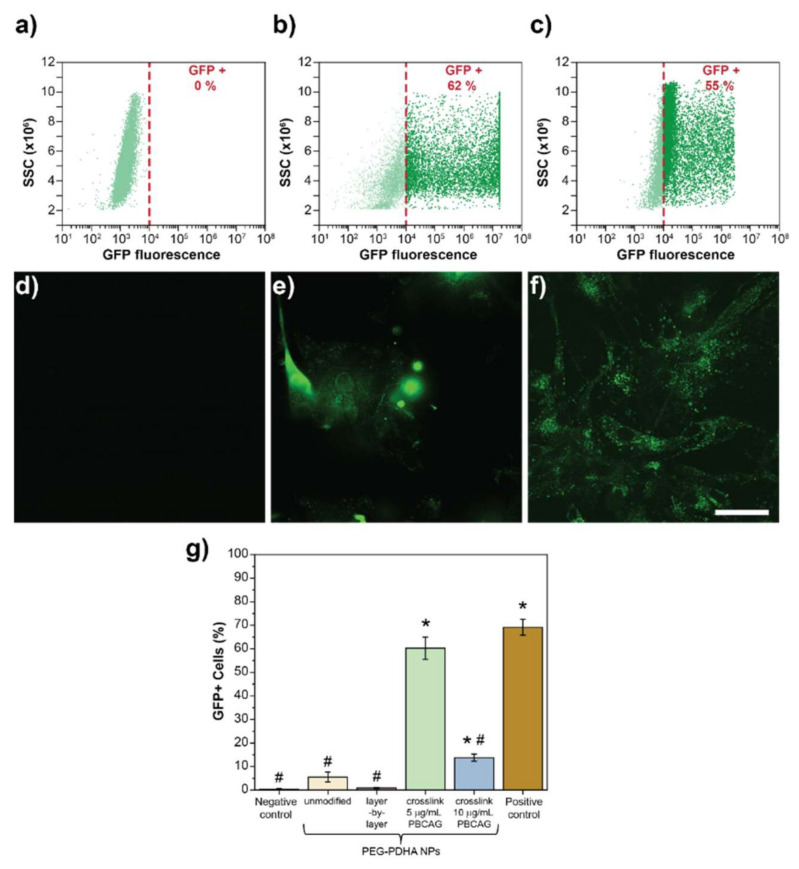
GFP expression on human glioblastoma (U87MG) cells as an indicator of PBCAG transfection. GFP expression on U87MG cells quantified by flow cytometry and their corresponding confocal microscopy image 72 h post-treatment for: (**a**,**d**) intact cells (negative control); (**b**,**e**) cells co-cultured with Lipofectamine 3000 carrying 5 μg of PBCAG (positive control); and (**c**,**f**) cells co-cultured with PEG-PDHA NPs carrying 5 μg of PBCAG. Scale bar = 50 μm. (**g**) Summarized GFP expression after PEG-PDHA nanoformulations and Lipofectamine 3000 transfection of PBCAG. Error bars represent standard deviation (*n* = 4). ANOVA test was performed to determine statistical significance. * indicates statistical difference with negative control and # indicates statistical difference with positive control (*p*-value < 0.05).

PEG-PDHA NPs were investigated for the intracellular delivery of PBCAG in U87MG cells. Transfection efficiency was characterized by GFP expression in U87MG as PBCAG plasmid is labeled with GFP. Flow cytometry was used to quantify transfection efficiency. Intact U87MG cells were used as the negative control. U87MG cells transfected with PBCAG using the commercially available Lipofectamine 3000 reagent was used as the positive control. The negative control served as the fluorescence threshold to differentiate between the GFP positive and negative cell populations. Intact U87MG cells do not express GFP or emit fluorescence in the GFP emission wavelength range (Figure 7a,d). PBCAG transfection with Lipofectamine 3000 resulted in ~62% of U87MG cells expressing GFP (Figure 7b,e). When U87MG cells were co-cultured with unmodified PEG-PDHA NPs only 7% of the cell population expressed GFP (Figure S5a). Low transfection efficiency from unmodified PEG-PDHA NPs is consistent with our release kinetics and cellular uptake studies. Although PEG-PDHA NPs are taken up by cells within 1 h of co-culturing, PBCAG burst release kinetics may impede efficient intracellular delivery of the plasmid. Consistent with our release studies, non-covalent stabilization of PEG-PDHA nanoformulation through the layer-by-layer technique was not efficient to release PBCAG and only less than 1% of the cell population expressed GFP (Figure S5b). Crosslinked PEG-PDHA NPs were capable to transfect U87MG cells with an efficiency of ~ 55% (Figure 7c,f). Figure 7g summarizes the cell transfection efficiency by the different nanoformulations. Although transfection efficiencies of crosslinked PEG-PDHA NPs and Lipofectamine 3000 are not statistically significantly different, U87MG cells transfected with Lipofectamine 3000 showed an abnormal spherical morphology with detectable cell detachment (Figure 7e), suggesting that cell integrity was compromised. From PEG-PDHA NPs formulations, the crosslinked NPs were the most efficient in transfecting PBCAG. Increasing the nanoformulation concentration from 5 to 10 μg mL^−1^ of PBCAG resulted in a consistent decrease of GFP positive U87MG cells. These results need to be further investigated.

### 3.7. PEG-PDHA NPs Cytotoxicity

**Figure 8:**
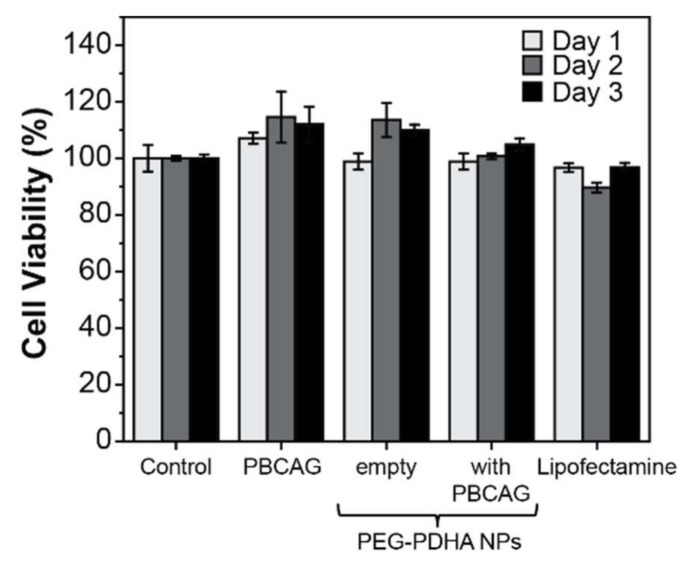
Cell viability of U87MG measured by 3-(4,5-dimethylthiazol-2-yl)-2,5-diphenyltetrazolium bromide (MTT) assay. Intact U87MG cells were used as the control. An ANOVA test was performed to determine statistical significance. No statistical significance was found between samples (*p*-value < 0.05).

The biocompatibility of PEG-PDHA NPs was studied in the U87MG cell line using MTT assay. Intact U87MG cells were used as the control. No cytotoxic effects were observed when U87MG cells were exposed to either naked PBCAG, empty PEG-PDHA NPs, or crosslinked PEG-PDHA NPs encapsulating PBCAG (Figure 8). Although previous studies have reported cytotoxic effects associated with Lipofectamine plasmid DNA delivery [46], we did not detect cytotoxicity in U87MG cells for PBCAG delivery using Lipofectamine 3000.

## 4. Discussion

We investigated the encapsulation of the gene editing tool PBCAG into a PEG-PDHA nanoformulation for its stable intracellular delivery. Our results suggest that PEG-PDHA forms a weak complex with PBCAG, which is expected as the complexation is occurring with a tertiary amine from the PDHA block. However, in previous studies, PDHA weak complexation was efficient for the intracellular delivery of relatively short (<4.8 kbp) DNA [47] and siRNA [37,42,43] plasmids. For larger plasmids such as PBCAG (~6.3 kbp), PEG-PDHA nanoformulations require further functionalization to slow down release kinetics and/or enhance intracellular uptake. It has been previously reported that unmodified PEG-PDHA NPs transfection efficiency for piggyBac transposon is less than 1%, and it can be increased by 3.4-fold when the nanoformulation is engineered with specific antibodies to enhance uptake [34]. Thus, we investigated the stabilization of PEG-PDHA NPs to enhance PBCAG intracellular delivery. Layer-by-layer surface engineering of PEG-PDHA NPs formed an extremely stable nanoformulation, from which release of PBCAG was not detected within 24 h. This result could be attributed to the electrostatic interaction between negatively charged PBCAG and positively charged PLL. Release of PBCAG from layer-by-layer engineered PEG-PDHA NPs will depend on the exfoliation of the multilayered coating, thus PBCAG will directly interact with PLL and PGA polymer layers [28,46]. PLL is one of the first cationic polymers investigated in non-viral gene delivery. It has been shown that the polyplexes formed by plasmid DNA and PLL are very stable [48,49], which could be hindering the release of PBCAG from the layer-by-layer nanoformulation. Crosslinking of the PEG-PDHA nanoformulation can slow down the release kinetics of PBCAG in PBS. For in vitro or in vivo intracellular plasmid delivery, release kinetics are expected to be enhanced, as other components, like proteins and enzymes, will interact with the nanoformulation and compromise its integrity. Thus, a slow-release kinetics profile in PBS is preferable to ensure potential success in intracellular delivery.

A vehicle for efficient gene delivery therapy must be capable to import the cargo into the cell nucleus, thus various targeting molecules have been used to ensure nanocarriers nuclear internalization [34,50]. Crosslinked PEG-PDHA NPs are able to translocate into the cell nucleus within a few hours of co-culturing and in the absence of a targeting moiety commending their potential success as nanocarriers for gene therapy. However, further studies are needed to elucidate the nuclear translocation mechanism of PEG-PDHA NPs. At pH 4, PEG-PDHA is protonated, which allows it to electrostatically encapsulate PBCAG [51]. At physiological pH, the PDHA from the block co-polymers forms a hydrophobic core protecting the plasmid from degradation [52]. Finally, when PEG-PDHA NPs enter the cell, the NPs dissociate at intracellular pH (<6), releasing the genetic material. We confirm GFP expression by flow cytometry and confocal microscopy. GFP is recruited by intracellular lipid bodies that are visualized in confocal microscopy. The recruitment of expressed proteins and plasmids by intracellular lipid bodies was observed before and it is specific to the cell line [53]. Although transfection efficiencies of crosslinked PEG-PDHA NPs and Lipofectamine 3000 are not statistically significantly different, a discrepancy in the cell integrity was observed when imaged. U87MG cells transfected with Lipofectamine 3000 showed an abnormal spherical morphology with detectable cell detachment, suggesting that cell integrity was compromised. Biocompatibility of Lipofectamine in U87MG cells is not consistent with our observations of cell integrity in cell transfection studies. This can be attributed to the nature of both MTT and U87MG cells. MTT correlates cell viability with metabolic activity, and the U87MG cell line is known to be capable of growing in 3D. Although Lipofectamine may be disturbing cell integrity, abnormal spherical or floating cells may still be functional. In fact, we have previously investigated the biocompatibility of Lipofectamine 3000 through a MTT assay in HEK293 cells, where we found that Lipofectamine 3000 is cytotoxic to HEK293 cells (60% cell viability) [46]. Further investigations are required to fully assess PEG-PDHA NPs and Lipofectamine biocompatibility.

## 5. Conclusions

Co-polymers from PBAEs represent a promising tool for the controllable and programmable non-viral delivery of gene-editing tools that could potentially overcome major challenges in the clinical translation of gene-editing therapy. The work presented here illustrates the versatility of PBAE co-polymers for chemical modification and emphasizes the importance of engineering non-viral vectors to gain control over cargo loading and release. The nanoformulation investigated in this work was designed to maintain stability under physiological conditions and to trigger the release of therapeutic agents upon intracellular pH change. This work proposes a facile and safe route to stabilize non-viral PBAE carriers for the intracellular delivery of gene-editing tools at efficiencies comparable to commercially available Lipofectamine 3000 reagent but not causing cytotoxic effects. Further investigations will require surface engineering with specific recognition functions to overcome biological barriers and their application using in vivo disease models.

## Supplementary Materials

The following are available online at https://www.mdpi.com/2306-5354/8/2/16/s1, Figure S1: PEG-PDHA block co-polymer characterization; Figure S2: PEG-PDHA NPs encapsulating PBCAG at a polymer/plasmid molar ratio of 0.36; Figure S3: Representative gel electrophoresis for the release of PBCAG at pH 4; Figure S4: Representative confocal microscopy images of U87MG cells co-cultured with PEG-PDHA NPs; Figure S5: GFP expression on U87MG cells as an indicator of PBCAG transfection for control samples.
