# Supplementary material for: Stabilization of Poly (β-Amino Ester) Nanoparticles for the Efficient Intracellular Delivery of PiggyBac Transposon
Source: Bioengineering (Basel). 2021 Jan 20;8(2):16. doi: 10.3390/bioengineering8020016 (PMC7909559; doi:10.3390/bioengineering8020016)
Supplement: Supplementary file 1 [file bioengineering-08-00016-s001.pdf]

*Supporting Information*

# Stabilization of Poly(b-amino ester) Nanoparticles for the Efficient Intracellular Delivery of PiggyBac Transposon

Tina Rodgers, Nicolas Muzzio, Caleb Watson and Gabriela Romero*

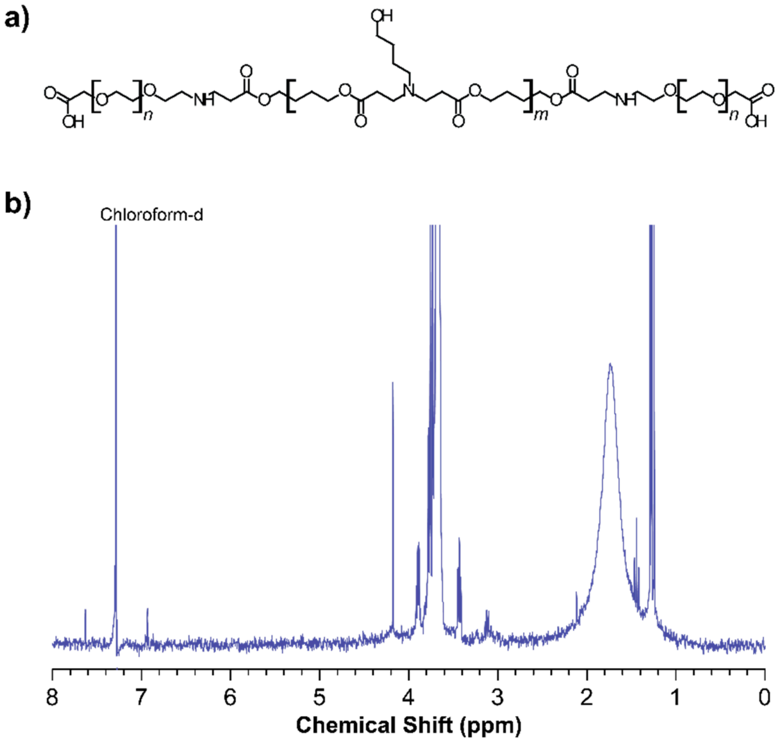


**Figure S1.** PEG-PDHA block co-polymer. a) Chemical structure of PEG-PDHA, and b) PEG-PDHA $^{1}$H-NMR spectrum in deuterated chloroform.

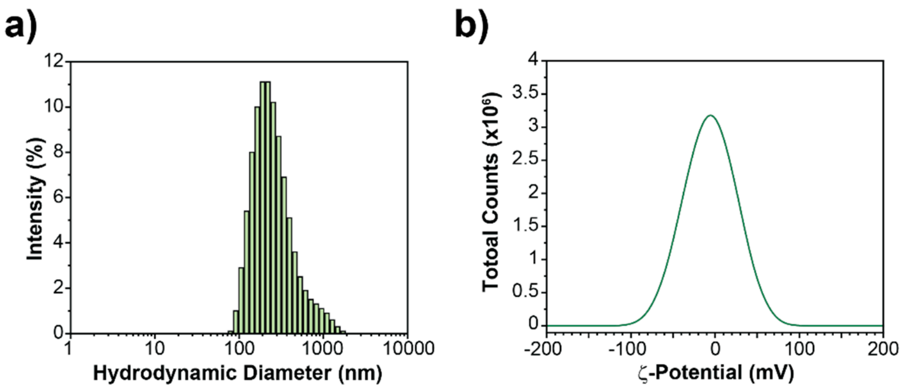


**Figure S2.** PEG-PDHA NPs encapsulating PBCAG at a polymer/plasmid molar ratio of 0.36. a) Hydrodynamic size distribution, and b) z-potential.

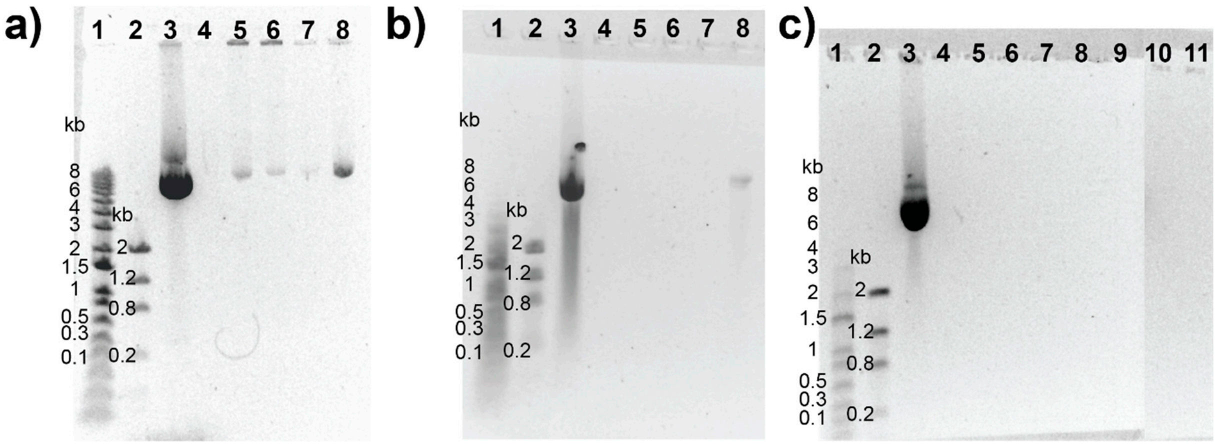


**Figure S3.** Representative gel electrophoresis for the release of PBCAG at pH 4 from: a) unmodified PEG-PDHA NPs, b) layer-by-layer engineered PEG-PDHA NPs, and c) crosslinked PEG-PDHA NPs. Well 1: 1 kb DNA ladder, well 2: low DNA mass ladder, well 3: PBCAG, well 4-11: PEG-PDHA NPs encapsulating PBCAG after 0, 0.5, 1, 3, 24, 48, 72, and 240 hours incubation at 37 °C respectively.

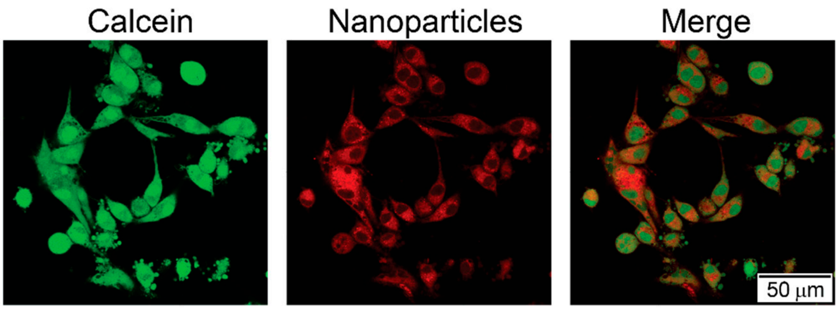


**Figure S4.** Representative confocal microscopy images of U87MG cells co-cultured with PEG-PDHA NPs. Live cells were stained with Calcein (green), and NPs were labeled with Rhodamine B (red).

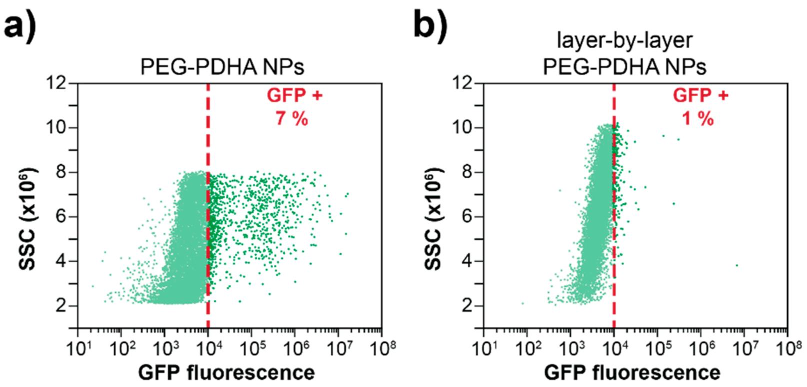


**Figure S5.** GFP expression on U87MG cells as an indicator of PBCAG transfection. GFP expression on U87MG cells quantified by flow cytometry for: a) unmodified PEG-PDHA NPs and b) PEG-PDHA NPs modified by layer-by-layer assembly.
